# 9-(5-Bromo-1*H*-indol-3-yl)-1,2,3,4,5,6,7,8,9,10-deca­hydro­acridine-1,8-dione dimethyl sulfoxide monosolvate

**DOI:** 10.1107/S1600536812045886

**Published:** 2012-11-10

**Authors:** Ahmed El-Khouly, Sema Öztürk Yildirim, Ray J. Butcher, Rahime Şimsek, Cihat Şafak

**Affiliations:** a Hacettepe University, Faculty of Pharmacy, Dept. of Pharmaceutical Chemistry, 06100 Sihhiye-Ankara, Turkey; b Department of Chemistry, Howard University, 525 College Street NW, Washington, DC 20059, USA; c Department of Physics, Faculty of Sciences, Erciyes University, 38039 Kayseri, Turkey

## Abstract

In the title compound, C_21_H_19_BrN_2_O_2_·C_2_H_6_OS, the indole ring system is essentially planar, with a maximum deviation of 0.050 (3) Å for the non-bridgehead C atom adjacent to the N atom. The two cyclo­hex-2-enone rings adopt half-chair conformations. An intra­molecular C—H⋯O hydrogen bond occurs. The solvent mol­ecule exhibits minor disorder of the S atom [site occupancies = 0.8153 (16) and 0.1847 (18)]. In the crystal, mol­ecules are linked by N—H⋯O hydrogen bonds, forming layers parallel to the *bc* plane.

## Related literature

For biological properties of acridines, including anti­bacterial, anti-parasitic, and anti­tumor activity, see: Biwersi *et al.* (1994 ▶); Wainwright (2001 ▶); Guetzoyan *et al.* (2007 ▶); Denny (2002 ▶); Luan *et al.* (2011 ▶). For recent studies showing that some acridine analogs having aryl and heteroaryl substituents at the ten position on the ring exert potassium-channel-modulating activiy, see: Şimşek *et al.* (2004 ▶), Berkan *et al.* (2002 ▶). For a description of the Cambridge Structural Database, see: Allen, (2002 ▶).
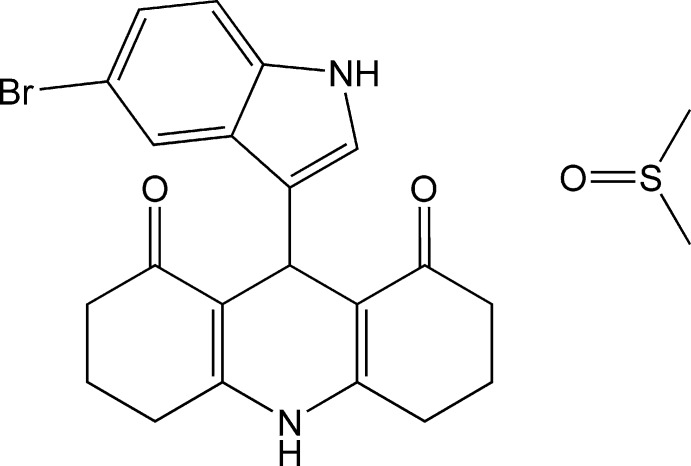


## Experimental

### 

#### Crystal data

C_21_H_19_BrN_2_O_2_·C_2_H_6_OS
*M* *_r_* = 489.42
Monoclinic, 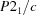

*a* = 9.1544 (4) Å
*b* = 18.9619 (8) Å
*c* = 12.9790 (5) Å
β = 105.623 (4)°
*V* = 2169.72 (16) Å^3^
*Z* = 4
Cu *K*α radiation
μ = 3.71 mm^−1^
*T* = 123 K
0.51 × 0.23 × 0.12 mm

#### Data collection

Agilent Xcalibur (Ruby, Gemini) diffractometer
Absorption correction: analytical [*CrysAlis PRO* (Agilent, 2011 ▶), using a multi-faceted crystal model (Clark & Reid, 1995 ▶)] *T*_min_ = 0.272, *T*_max_ = 0.721
14006 measured reflections
4444 independent reflections
4183 reflections with *I* > 2σ(*I*)
*R*_int_ = 0.041

#### Refinement

*R*[*F* ^2^ > 2σ(*F* ^2^)] = 0.052
*wR*(*F* ^2^) = 0.137
*S* = 1.06
4444 reflections
290 parameters
6 restraints
H-atom parameters constrained
Δρ_max_ = 1.96 e Å^−3^
Δρ_min_ = −1.35 e Å^−3^

### 

Data collection: *CrysAlis PRO* (Agilent, 2011 ▶); cell refinement: *CrysAlis PRO*; data reduction: *CrysAlis PRO*; program(s) used to solve structure: *SHELXS97* (Sheldrick, 2008 ▶); program(s) used to refine structure: *SHELXL97* (Sheldrick, 2008 ▶); molecular graphics: *SHELXTL* (Sheldrick, 2008 ▶); software used to prepare material for publication: *SHELXTL*.

## Supplementary Material

Crystal structure: contains datablock(s) I, global. DOI: 10.1107/S1600536812045886/hg5266sup1.cif

Structure factors: contains datablock(s) I. DOI: 10.1107/S1600536812045886/hg5266Isup2.hkl

Supplementary material file. DOI: 10.1107/S1600536812045886/hg5266Isup3.cml

Additional supplementary materials:  crystallographic information; 3D view; checkCIF report

## Figures and Tables

**Table 1:** Hydrogen-bond geometry (Å, °)

*D*—H⋯*A*	*D*—H	H⋯*A*	*D*⋯*A*	*D*—H⋯*A*
C16—H16*A*⋯O1	0.95	2.50	3.252 (3)	136
N1—H1*A*⋯O2^i^	0.88	2.03	2.901 (3)	173
N2—H2*C*⋯O100^ii^	0.88	2.10	2.920 (3)	154
N2—H2*C*⋯O100^iii^	0.88	2.52	3.038 (3)	118

Symmetry codes: (i) 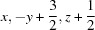; (ii) 
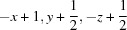
; (iii) 
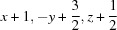
.

**Fig. 1.**
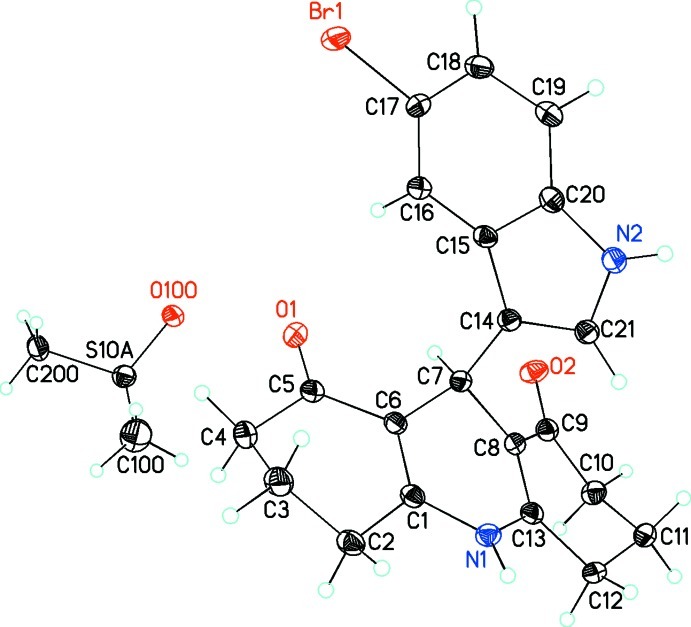
The molecular structure of (I) with 30% probability ellipsoids for non-H atoms. Only major disordered component for the solvent molecule is shown.

**Fig. 2.**
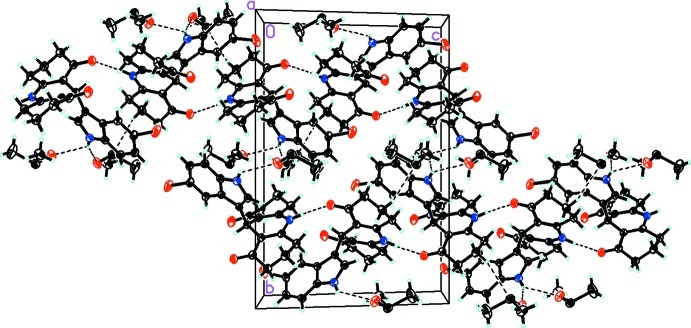
The packing of (I), viewed down a axis, showing molecules are linked into plane parallel to bc plane. Only major disordered component for the solvent molecule is shown and hydrogen bonds are shown as dashed lines.

## supplementary crystallographic information

### Comment

Acridines display abroad range of biological properties including antibacterial, anti-parasitic, and antitumor activities (Biwersi *et al.*, 1994; Wainwright, 2001;Guetzoyan *et al.*, 2007; Denny, 2002; Luan *et al.*, 2011). Furthermore, the indole moiety also has a wide range of biological activities which may enhance the acridine ring properties. Recent studies show that some acridine analogs having aryl and heteroaryl substituents on their ten position on the ring exert potassium channel modulating activities (Şimşek *et al.*, 2004; Berkan *et al.*, 2002).

The title acridine compound contains (Fig. 1), 5-bromo-3-methyl-1*H*-indole connected to the 3,4,6,7,9,10-hexahydro-2H,5*H*-acridine-1,8-dione system and the disordered dimethyl sulfoxide solvent molecule. The Br—C bond distance [1.910 (3) Å] is in the normal range (Allen, 2002). The 1-H indole ring system is essentially planar with a maximum deviation of -0.050 (3) Å for atom C21. The 1*H*-indol ring system forms a dihedral angle of 22.40 (12) ° with the 1,4-dihydro-pyridine ring (N1/C1/C6—C8/C13). The two cyclohex-2-enone rings (C1—C6 and C8—C13) adopt half chair conformations with C3 atom 0.345 (3) Å and C11 atom 0.335 (3) Å out of the mean-plane formed by the remaining ring atoms. The solvent molecule exhibits minor disorder of its S atom [site occupancies = 0.8153 (16) and 0.1847 (18)]. In the crystal, molecules are linked by C—H···O and N—H···O hydrogen bonds (Tab. 1 & Fig. 2).

### Experimental

A mixture of 5-bromoindole-3-carbaldehyde (1.0 mmol), 1,3-cyclohexanedione (2.0 mmol), ammonium acetate (5.0 mmol) was dissolved in 5 ml of methanol and refluxed until the reaction was completed (monitored by TLC). The forming precipitate was filtered off and crystallized from ethanol. Crystals were grown by slow evaporation of an dimethyl sulfoxide/methanol mixed solution.

### Refinement

All disordered components were subjected to rigid bond and similarity restraints and all major and minor disordered components were refined anisotropically. Hydrogen atoms were positioned geometrically [C—H = 0.95–1.00 Å; N—H = 0.88 Å] and refined using a riding model, with *U*_iso_(H) = 1.2 *U*_eq_(C, N) or 1.5 *U*_eq_(C_methyl_). A rotating-group model was applied for methyl groups.

### Crystal data

**Table:**

C_21_H_19_BrN_2_O_2_·C_2_H_6_OS	*F*(000) = 1008
*M**_r_* = 489.42	*D*_x_ = 1.498 Mg m^−^^3^
Monoclinic, *P*2_1_/*c*	Cu *K*α radiation, λ = 1.54184 Å
Hall symbol: -P 2ybc	Cell parameters from 7435 reflections
*a* = 9.1544 (4) Å	θ = 3.5–75.5°
*b* = 18.9619 (8) Å	µ = 3.71 mm^−^^1^
*c* = 12.9790 (5) Å	*T* = 123 K
β = 105.623 (4)°	Prism, light-yellow
*V* = 2169.72 (16) Å^3^	0.51 × 0.23 × 0.12 mm
*Z* = 4	

### Data collection

**Table:**

Agilent Xcalibur (Ruby, Gemini) diffractometer	4444 independent reflections
Radiation source: Enhance (Cu) X-ray Source	4183 reflections with *I* > 2σ(*I*)
Graphite monochromator	*R*_int_ = 0.041
Detector resolution: 10.5081 pixels mm^-1^	θ_max_ = 75.7°, θ_min_ = 4.2°
ω scans	*h* = −10→11
Absorption correction: analytical [*CrysAlis PRO* (Agilent, 2011), using a multi-faceted crystal model (Clark & Reid, 1995)]	*k* = −20→23
*T*_min_ = 0.272, *T*_max_ = 0.721	*l* = −15→16
14006 measured reflections	

### Refinement

**Table:**

Refinement on *F*^2^	Primary atom site location: structure-invariant direct methods
Least-squares matrix: full	Secondary atom site location: difference Fourier map
*R*[*F*^2^ > 2σ(*F*^2^)] = 0.052	Hydrogen site location: inferred from neighbouring sites
*wR*(*F*^2^) = 0.137	H-atom parameters constrained
*S* = 1.06	*w* = 1/[σ^2^(*F*_o_^2^) + (0.0699*P*)^2^ + 3.3586*P*] where *P* = (*F*_o_^2^ + 2*F*_c_^2^)/3
4444 reflections	(Δ/σ)_max_ = 0.001
290 parameters	Δρ_max_ = 1.96 e Å^−^^3^
6 restraints	Δρ_min_ = −1.35 e Å^−^^3^

### Special details

**Table:**

Geometry. All e.s.d.'s (except the e.s.d. in the dihedral angle between two l.s. planes) are estimated using the full covariance matrix. The cell e.s.d.'s are taken into account individually in the estimation of e.s.d.'s in distances, angles and torsion angles; correlations between e.s.d.'s in cell parameters are only used when they are defined by crystal symmetry. An approximate (isotropic) treatment of cell e.s.d.'s is used for estimating e.s.d.'s involving l.s. planes.
Refinement. Refinement of *F*^2^ against ALL reflections. The weighted *R*-factor *wR* and goodness of fit *S* are based on *F*^2^, conventional *R*-factors *R* are based on *F*, with *F* set to zero for negative *F*^2^. The threshold expression of *F*^2^ > σ(*F*^2^) is used only for calculating *R*-factors(gt) *etc*. and is not relevant to the choice of reflections for refinement. *R*-factors based on *F*^2^ are statistically about twice as large as those based on *F*, and *R*- factors based on ALL data will be even larger.

### Fractional atomic coordinates and isotropic or equivalent isotropic displacement parameters (Å^2^)

**Table:**

	*x*	*y*	*z*	*U*_iso_*/*U*_eq_	Occ. (<1)
S10A	−0.13796 (9)	0.48345 (4)	0.17928 (7)	0.03482 (18)	0.8153 (16)
O100	−0.0432 (3)	0.51319 (16)	0.1107 (2)	0.0382 (7)	0.8153 (16)
C100	−0.0885 (11)	0.5268 (4)	0.3027 (4)	0.0648 (14)	0.8153 (16)
H10C	0.0119	0.5110	0.3443	0.097*	0.8153 (16)
H10D	−0.1634	0.5160	0.3419	0.097*	0.8153 (16)
H10E	−0.0863	0.5778	0.2910	0.097*	0.8153 (16)
C200	−0.3247 (4)	0.5167 (3)	0.1308 (6)	0.0412 (9)	0.8153 (16)
H20A	−0.3729	0.4955	0.0611	0.062*	0.8153 (16)
H20B	−0.3210	0.5680	0.1231	0.062*	0.8153 (16)
H20C	−0.3835	0.5050	0.1814	0.062*	0.8153 (16)
S10B	−0.1368 (4)	0.54694 (19)	0.1798 (3)	0.03482 (18)	0.1847 (16)
O101	−0.0449 (18)	0.4988 (9)	0.1276 (13)	0.0382 (7)	0.1847 (16)
C101	−0.096 (5)	0.517 (2)	0.3127 (12)	0.0648 (14)	0.1847 (16)
H10F	0.0071	0.4981	0.3344	0.097*	0.1847 (16)
H10G	−0.1685	0.4803	0.3183	0.097*	0.1847 (16)
H10H	−0.1043	0.5567	0.3596	0.097*	0.1847 (16)
C201	−0.3306 (14)	0.5260 (18)	0.132 (3)	0.0412 (9)	0.1847 (16)
H20D	−0.3710	0.5458	0.0604	0.062*	0.1847 (16)
H20E	−0.3858	0.5458	0.1805	0.062*	0.1847 (16)
H20F	−0.3430	0.4746	0.1291	0.062*	0.1847 (16)
Br1	0.25418 (3)	0.905937 (19)	0.00832 (2)	0.04525 (11)	
O1	0.2313 (2)	0.77721 (11)	0.33651 (15)	0.0352 (4)	
O2	0.7181 (2)	0.65871 (10)	0.37707 (14)	0.0340 (4)	
N1	0.6163 (2)	0.78876 (11)	0.65949 (16)	0.0260 (4)	
H1A	0.6514	0.8073	0.7235	0.031*	
N2	0.7825 (2)	0.92633 (11)	0.39600 (17)	0.0269 (4)	
H2C	0.8614	0.9546	0.4157	0.032*	
C1	0.4715 (3)	0.80553 (12)	0.59876 (19)	0.0243 (5)	
C2	0.3651 (3)	0.83425 (14)	0.6587 (2)	0.0296 (5)	
H2A	0.4191	0.8691	0.7123	0.036*	
H2B	0.3301	0.7954	0.6972	0.036*	
C3	0.2284 (3)	0.86936 (15)	0.5820 (2)	0.0365 (6)	
H3A	0.1516	0.8807	0.6205	0.044*	
H3B	0.2604	0.9140	0.5551	0.044*	
C4	0.1589 (3)	0.82086 (16)	0.4883 (2)	0.0364 (6)	
H4A	0.1152	0.7791	0.5149	0.044*	
H4B	0.0753	0.8460	0.4372	0.044*	
C5	0.2730 (3)	0.79654 (13)	0.4305 (2)	0.0275 (5)	
C6	0.4318 (3)	0.79381 (12)	0.49155 (19)	0.0239 (5)	
C7	0.5513 (2)	0.77218 (12)	0.43652 (18)	0.0225 (4)	
H7A	0.5026	0.7423	0.3730	0.027*	
C8	0.6706 (3)	0.72888 (12)	0.51402 (18)	0.0235 (5)	
C9	0.7466 (3)	0.67249 (13)	0.4734 (2)	0.0265 (5)	
C10	0.8583 (3)	0.62845 (14)	0.5553 (2)	0.0305 (5)	
H10A	0.9300	0.6052	0.5210	0.037*	
H10B	0.8032	0.5912	0.5830	0.037*	
C11	0.9464 (3)	0.67432 (14)	0.6478 (2)	0.0303 (5)	
H11A	1.0056	0.7101	0.6207	0.036*	
H11B	1.0183	0.6446	0.7008	0.036*	
C12	0.8380 (3)	0.71116 (13)	0.70158 (19)	0.0270 (5)	
H12A	0.8002	0.6766	0.7455	0.032*	
H12B	0.8934	0.7486	0.7498	0.032*	
C13	0.7059 (3)	0.74323 (12)	0.62042 (19)	0.0241 (5)	
C14	0.6227 (3)	0.83598 (12)	0.39910 (18)	0.0230 (4)	
C15	0.5613 (3)	0.87732 (12)	0.30416 (18)	0.0235 (4)	
C16	0.4347 (3)	0.86999 (13)	0.21609 (19)	0.0261 (5)	
H16A	0.3642	0.8327	0.2122	0.031*	
C17	0.4162 (3)	0.91920 (14)	0.1349 (2)	0.0296 (5)	
C18	0.5129 (3)	0.97717 (14)	0.1398 (2)	0.0300 (5)	
H18A	0.4911	1.0114	0.0843	0.036*	
C19	0.6399 (3)	0.98455 (13)	0.2253 (2)	0.0276 (5)	
H19A	0.7078	1.0229	0.2291	0.033*	
C20	0.6650 (3)	0.93375 (13)	0.30590 (19)	0.0253 (5)	
C21	0.7575 (3)	0.86762 (13)	0.45088 (19)	0.0253 (5)	
H21A	0.8242	0.8512	0.5157	0.030*	

### Atomic displacement parameters (Å^2^)

**Table:**

	*U*^11^	*U*^22^	*U*^33^	*U*^12^	*U*^13^	*U*^23^
S10A	0.0375 (4)	0.0344 (4)	0.0344 (4)	0.0084 (3)	0.0128 (3)	0.0046 (3)
O100	0.0318 (9)	0.0508 (17)	0.0359 (14)	0.0114 (11)	0.0159 (9)	0.0126 (11)
C100	0.058 (2)	0.093 (4)	0.0423 (19)	0.008 (2)	0.0118 (19)	−0.023 (2)
C200	0.0305 (13)	0.045 (2)	0.0518 (17)	0.0033 (13)	0.0173 (13)	0.0110 (16)
S10B	0.0375 (4)	0.0344 (4)	0.0344 (4)	0.0084 (3)	0.0128 (3)	0.0046 (3)
O101	0.0318 (9)	0.0508 (17)	0.0359 (14)	0.0114 (11)	0.0159 (9)	0.0126 (11)
C101	0.058 (2)	0.093 (4)	0.0423 (19)	0.008 (2)	0.0118 (19)	−0.023 (2)
C201	0.0305 (13)	0.045 (2)	0.0518 (17)	0.0033 (13)	0.0173 (13)	0.0110 (16)
Br1	0.03879 (18)	0.0606 (2)	0.02995 (16)	−0.00943 (13)	−0.00175 (13)	0.01575 (12)
O1	0.0266 (8)	0.0499 (11)	0.0276 (9)	0.0020 (8)	0.0049 (7)	0.0038 (8)
O2	0.0406 (10)	0.0372 (10)	0.0242 (8)	0.0118 (8)	0.0085 (7)	−0.0004 (7)
N1	0.0283 (9)	0.0290 (10)	0.0209 (9)	0.0015 (8)	0.0071 (8)	−0.0011 (7)
N2	0.0255 (9)	0.0284 (10)	0.0278 (10)	−0.0025 (8)	0.0089 (8)	−0.0006 (8)
C1	0.0260 (10)	0.0227 (10)	0.0262 (11)	0.0004 (8)	0.0102 (9)	0.0033 (8)
C2	0.0329 (12)	0.0307 (12)	0.0293 (12)	0.0062 (10)	0.0153 (10)	0.0024 (9)
C3	0.0352 (13)	0.0416 (14)	0.0368 (13)	0.0128 (11)	0.0170 (11)	0.0044 (11)
C4	0.0254 (11)	0.0499 (15)	0.0364 (14)	0.0071 (11)	0.0126 (10)	0.0076 (12)
C5	0.0260 (11)	0.0303 (12)	0.0273 (11)	0.0022 (9)	0.0090 (9)	0.0074 (9)
C6	0.0233 (10)	0.0252 (11)	0.0250 (11)	0.0019 (8)	0.0096 (9)	0.0042 (8)
C7	0.0220 (10)	0.0248 (10)	0.0209 (10)	0.0014 (8)	0.0062 (8)	0.0009 (8)
C8	0.0214 (10)	0.0255 (11)	0.0239 (11)	0.0001 (8)	0.0065 (8)	0.0028 (9)
C9	0.0257 (11)	0.0279 (11)	0.0266 (11)	0.0018 (9)	0.0082 (9)	0.0018 (9)
C10	0.0297 (11)	0.0318 (12)	0.0298 (12)	0.0095 (10)	0.0077 (10)	0.0017 (10)
C11	0.0241 (11)	0.0369 (13)	0.0291 (12)	0.0049 (10)	0.0059 (9)	0.0056 (10)
C12	0.0267 (11)	0.0321 (12)	0.0213 (10)	0.0016 (9)	0.0048 (9)	0.0042 (9)
C13	0.0245 (10)	0.0243 (10)	0.0246 (11)	0.0000 (9)	0.0083 (9)	0.0039 (8)
C14	0.0242 (10)	0.0252 (11)	0.0210 (10)	0.0034 (8)	0.0086 (8)	−0.0006 (8)
C15	0.0259 (10)	0.0239 (10)	0.0233 (10)	0.0037 (9)	0.0115 (9)	0.0014 (8)
C16	0.0268 (11)	0.0272 (11)	0.0246 (11)	0.0003 (9)	0.0077 (9)	0.0028 (9)
C17	0.0256 (11)	0.0388 (13)	0.0224 (11)	0.0013 (10)	0.0032 (9)	0.0050 (10)
C18	0.0348 (12)	0.0311 (12)	0.0267 (11)	0.0038 (10)	0.0129 (10)	0.0065 (9)
C19	0.0314 (11)	0.0251 (11)	0.0305 (12)	−0.0002 (9)	0.0154 (10)	0.0025 (9)
C20	0.0259 (10)	0.0254 (11)	0.0272 (11)	0.0015 (9)	0.0113 (9)	−0.0022 (9)
C21	0.0248 (10)	0.0288 (11)	0.0235 (10)	0.0022 (9)	0.0086 (9)	0.0000 (9)

### Geometric parameters (Å, º)

**Table:**

S10A—O100	1.510 (3)	C3—H3B	0.9900
S10A—C100	1.748 (6)	C4—C5	1.512 (4)
S10A—C200	1.771 (4)	C4—H4A	0.9900
C100—H10C	0.9800	C4—H4B	0.9900
C100—H10D	0.9800	C5—C6	1.457 (3)
C100—H10E	0.9800	C6—C7	1.515 (3)
C200—H20A	0.9800	C7—C8	1.512 (3)
C200—H20B	0.9800	C7—C14	1.515 (3)
C200—H20C	0.9800	C7—H7A	1.0000
S10B—O101	1.519 (11)	C8—C13	1.358 (3)
S10B—C101	1.757 (11)	C8—C9	1.450 (3)
S10B—C201	1.760 (10)	C9—C10	1.513 (3)
C101—H10F	0.9800	C10—C11	1.525 (4)
C101—H10G	0.9800	C10—H10A	0.9900
C101—H10H	0.9800	C10—H10B	0.9900
C201—H20D	0.9800	C11—C12	1.527 (3)
C201—H20E	0.9800	C11—H11A	0.9900
C201—H20F	0.9800	C11—H11B	0.9900
Br1—C17	1.910 (3)	C12—C13	1.502 (3)
O1—C5	1.232 (3)	C12—H12A	0.9900
O2—C9	1.235 (3)	C12—H12B	0.9900
N1—C13	1.378 (3)	C14—C21	1.374 (3)
N1—C1	1.385 (3)	C14—C15	1.441 (3)
N1—H1A	0.8800	C15—C16	1.398 (3)
N2—C20	1.366 (3)	C15—C20	1.426 (3)
N2—C21	1.374 (3)	C16—C17	1.383 (3)
N2—H2C	0.8800	C16—H16A	0.9500
C1—C6	1.358 (3)	C17—C18	1.402 (4)
C1—C2	1.503 (3)	C18—C19	1.382 (4)
C2—C3	1.526 (4)	C18—H18A	0.9500
C2—H2A	0.9900	C19—C20	1.394 (3)
C2—H2B	0.9900	C19—H19A	0.9500
C3—C4	1.520 (4)	C21—H21A	0.9500
C3—H3A	0.9900		
			
O100—S10A—C100	108.4 (3)	C8—C7—H7A	108.8
O100—S10A—C200	108.1 (3)	C6—C7—H7A	108.8
C100—S10A—C200	98.3 (4)	C14—C7—H7A	108.8
O101—S10B—C101	104.4 (16)	C13—C8—C9	120.7 (2)
O101—S10B—C201	109.5 (14)	C13—C8—C7	119.9 (2)
C101—S10B—C201	101 (2)	C9—C8—C7	119.3 (2)
S10B—C101—H10F	109.5	O2—C9—C8	122.2 (2)
S10B—C101—H10G	109.5	O2—C9—C10	120.9 (2)
H10F—C101—H10G	109.5	C8—C9—C10	116.8 (2)
S10B—C101—H10H	109.5	C9—C10—C11	110.6 (2)
H10F—C101—H10H	109.5	C9—C10—H10A	109.5
H10G—C101—H10H	109.5	C11—C10—H10A	109.5
S10B—C201—H20D	109.5	C9—C10—H10B	109.5
S10B—C201—H20E	109.5	C11—C10—H10B	109.5
H20D—C201—H20E	109.5	H10A—C10—H10B	108.1
S10B—C201—H20F	109.5	C10—C11—C12	110.5 (2)
H20D—C201—H20F	109.5	C10—C11—H11A	109.6
H20E—C201—H20F	109.5	C12—C11—H11A	109.6
C13—N1—C1	120.5 (2)	C10—C11—H11B	109.6
C13—N1—H1A	119.7	C12—C11—H11B	109.6
C1—N1—H1A	119.7	H11A—C11—H11B	108.1
C20—N2—C21	108.9 (2)	C13—C12—C11	111.3 (2)
C20—N2—H2C	125.5	C13—C12—H12A	109.4
C21—N2—H2C	125.5	C11—C12—H12A	109.4
C6—C1—N1	119.7 (2)	C13—C12—H12B	109.4
C6—C1—C2	123.9 (2)	C11—C12—H12B	109.4
N1—C1—C2	116.3 (2)	H12A—C12—H12B	108.0
C1—C2—C3	110.6 (2)	C8—C13—N1	119.8 (2)
C1—C2—H2A	109.5	C8—C13—C12	123.9 (2)
C3—C2—H2A	109.5	N1—C13—C12	116.2 (2)
C1—C2—H2B	109.5	C21—C14—C15	105.8 (2)
C3—C2—H2B	109.5	C21—C14—C7	126.8 (2)
H2A—C2—H2B	108.1	C15—C14—C7	127.3 (2)
C4—C3—C2	110.5 (2)	C16—C15—C20	119.3 (2)
C4—C3—H3A	109.5	C16—C15—C14	133.7 (2)
C2—C3—H3A	109.5	C20—C15—C14	106.9 (2)
C4—C3—H3B	109.5	C17—C16—C15	117.4 (2)
C2—C3—H3B	109.5	C17—C16—H16A	121.3
H3A—C3—H3B	108.1	C15—C16—H16A	121.3
C5—C4—C3	112.7 (2)	C16—C17—C18	123.2 (2)
C5—C4—H4A	109.1	C16—C17—Br1	118.29 (19)
C3—C4—H4A	109.1	C18—C17—Br1	118.48 (19)
C5—C4—H4B	109.1	C19—C18—C17	120.0 (2)
C3—C4—H4B	109.1	C19—C18—H18A	120.0
H4A—C4—H4B	107.8	C17—C18—H18A	120.0
O1—C5—C6	121.7 (2)	C18—C19—C20	117.8 (2)
O1—C5—C4	120.7 (2)	C18—C19—H19A	121.1
C6—C5—C4	117.5 (2)	C20—C19—H19A	121.1
C1—C6—C5	120.2 (2)	N2—C20—C19	130.2 (2)
C1—C6—C7	120.0 (2)	N2—C20—C15	107.7 (2)
C5—C6—C7	119.7 (2)	C19—C20—C15	122.1 (2)
C8—C7—C6	108.48 (19)	N2—C21—C14	110.7 (2)
C8—C7—C14	110.51 (19)	N2—C21—H21A	124.6
C6—C7—C14	111.30 (19)	C14—C21—H21A	124.6
			
C13—N1—C1—C6	−18.3 (3)	C7—C8—C13—N1	10.9 (3)
C13—N1—C1—C2	159.9 (2)	C9—C8—C13—C12	9.5 (4)
C6—C1—C2—C3	−17.9 (3)	C7—C8—C13—C12	−170.2 (2)
N1—C1—C2—C3	164.0 (2)	C1—N1—C13—C8	17.0 (3)
C1—C2—C3—C4	49.7 (3)	C1—N1—C13—C12	−161.9 (2)
C2—C3—C4—C5	−54.8 (3)	C11—C12—C13—C8	12.8 (3)
C3—C4—C5—O1	−156.3 (2)	C11—C12—C13—N1	−168.3 (2)
C3—C4—C5—C6	26.5 (3)	C8—C7—C14—C21	−22.8 (3)
N1—C1—C6—C5	166.7 (2)	C6—C7—C14—C21	97.8 (3)
C2—C1—C6—C5	−11.4 (4)	C8—C7—C14—C15	159.4 (2)
N1—C1—C6—C7	−8.5 (3)	C6—C7—C14—C15	−80.0 (3)
C2—C1—C6—C7	173.4 (2)	C21—C14—C15—C16	174.8 (3)
O1—C5—C6—C1	−170.3 (2)	C7—C14—C15—C16	−7.1 (4)
C4—C5—C6—C1	6.9 (3)	C21—C14—C15—C20	−1.1 (3)
O1—C5—C6—C7	4.9 (4)	C7—C14—C15—C20	177.1 (2)
C4—C5—C6—C7	−177.9 (2)	C20—C15—C16—C17	−0.7 (3)
C1—C6—C7—C8	32.0 (3)	C14—C15—C16—C17	−176.1 (2)
C5—C6—C7—C8	−143.2 (2)	C15—C16—C17—C18	−3.5 (4)
C1—C6—C7—C14	−89.8 (3)	C15—C16—C17—Br1	174.75 (18)
C5—C6—C7—C14	95.0 (2)	C16—C17—C18—C19	4.6 (4)
C6—C7—C8—C13	−33.3 (3)	Br1—C17—C18—C19	−173.56 (19)
C14—C7—C8—C13	89.0 (3)	C17—C18—C19—C20	−1.4 (4)
C6—C7—C8—C9	147.0 (2)	C21—N2—C20—C19	−179.6 (2)
C14—C7—C8—C9	−90.7 (3)	C21—N2—C20—C15	0.1 (3)
C13—C8—C9—O2	−179.2 (2)	C18—C19—C20—N2	177.0 (2)
C7—C8—C9—O2	0.5 (4)	C18—C19—C20—C15	−2.7 (4)
C13—C8—C9—C10	3.6 (3)	C16—C15—C20—N2	−176.0 (2)
C7—C8—C9—C10	−176.7 (2)	C14—C15—C20—N2	0.6 (3)
O2—C9—C10—C11	145.2 (2)	C16—C15—C20—C19	3.8 (3)
C8—C9—C10—C11	−37.5 (3)	C14—C15—C20—C19	−179.6 (2)
C9—C10—C11—C12	58.8 (3)	C20—N2—C21—C14	−0.9 (3)
C10—C11—C12—C13	−46.2 (3)	C15—C14—C21—N2	1.2 (3)
C9—C8—C13—N1	−169.4 (2)	C7—C14—C21—N2	−177.0 (2)

### Hydrogen-bond geometry (Å, º)

**Table:**

*D*—H···*A*	*D*—H	H···*A*	*D*···*A*	*D*—H···*A*
C7—H7*A*···O1	1.00	2.49	2.869 (3)	102
C16—H16*A*···O1	0.95	2.50	3.252 (3)	136
N1—H1*A*···O2^i^	0.88	2.03	2.901 (3)	173
N2—H2*C*···O100^ii^	0.88	2.10	2.920 (3)	154
N2—H2*C*···O100^iii^	0.88	2.52	3.038 (3)	118

Symmetry codes: (i) *x*, −*y*+3/2, *z*+1/2; (ii) −*x*+1, *y*+1/2, −*z*+1/2; (iii) *x*+1, −*y*+3/2, *z*+1/2.
